# Supplementary material for: STING induces early IFN-β in the liver and constrains myeloid cell-mediated dissemination of murine cytomegalovirus
Source: Nat Commun. 2019 Jun 27;10:2830. doi: 10.1038/s41467-019-10863-0 (PMC6597531; doi:10.1038/s41467-019-10863-0)
Supplement: Supplementary file 1 — Supplementary Information [file 41467_2019_10863_MOESM1_ESM.pdf]

STING induces early IFN-β in the liver and constrains myeloid cell-mediated dissemination of murine cytomegalovirus

Tegtmeyer and Spanier et al.

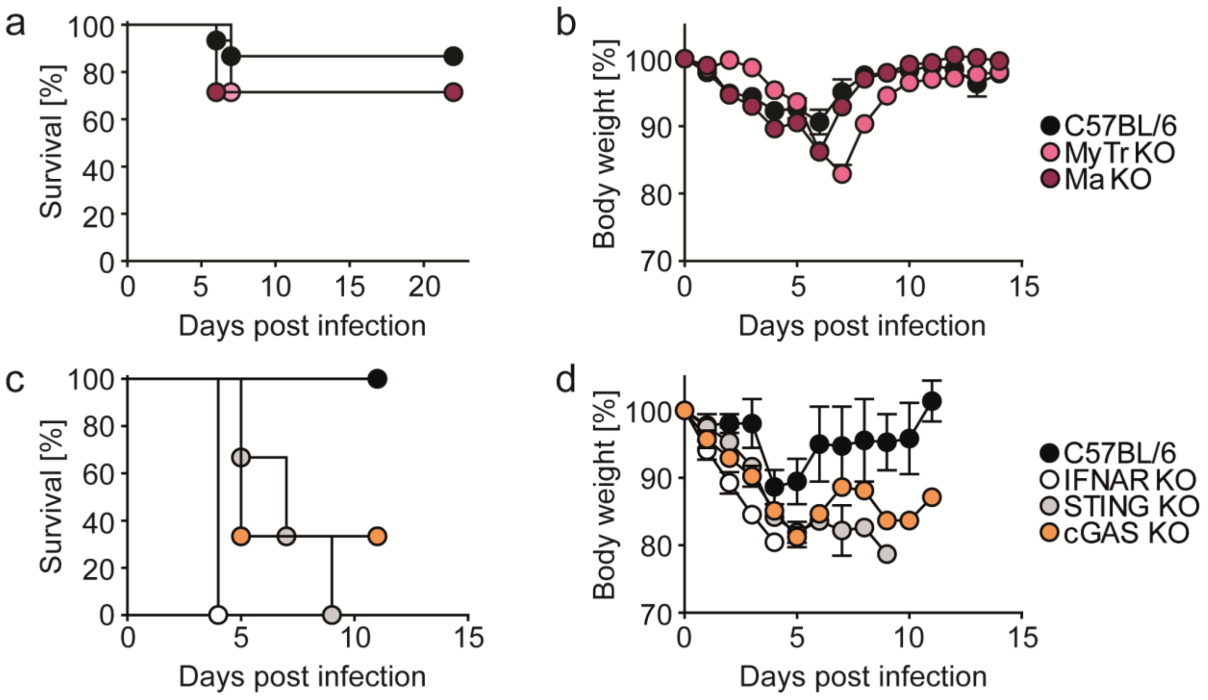

Supplementary Figure 1: cGAS KO and STING KO mice show enhanced susceptibility to lethal vaccinia virus infection.

C57BL/6 (*n* = 15), MyTr KO (*Myd88*$^{-/-}$*Trif*$^{-/-}$, *n* = 14), and Ma KO (*Mavs*$^{-/-}$, *n* = 14) mice were i.v. infected with 5×10^5 pfu MCMV Δ*m157* and **a** survival as well as **b** body weight was monitored daily. Survival and body weight of MyTr KO and Ma KO mice were analyzed in the same experiments as shown in Figure 1. Therefore, the depicted C57BL/6 control data are the same ones as in Figure 1. Data represent at least two independently performed experiments. C57BL/6 (*n* = 2), IFNAR KO (*Ifnar*$^{-/-}$) (*n* = 2), STING KO (*Tmem173*$^{-/-}$, *n* = 3), and cGAS KO (*Cgas*$^{-/-}$, *n* = 3) mice were i.v. infected with 2×10^6 pfu vaccinia virus and **c** survival as well as **d** body weight was monitored daily. In case body weight decreased by more than 20% of the initial body weight, or when the overall health status was dramatically impaired, mice were sacrificed. Data were retrieved in one experiment. Error bars indicate mean ± s.e.m. Source Data are provided as a Source Data file.

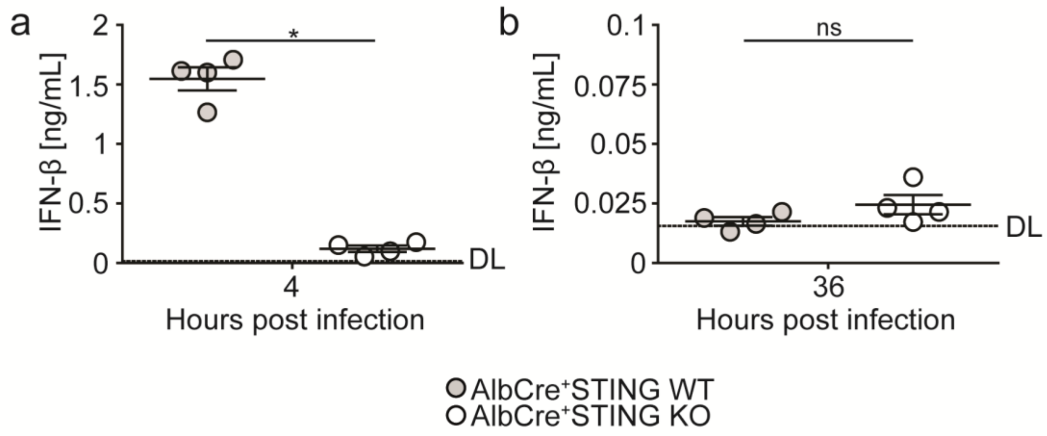

Supplementary Figure 2: After WT MCMV infection STING signaling is important for the induction of early serum IFN-β responses.

AlbCre^{+}STING WT (*AlbCre*$^{+/-}$*Tmem173*$^{wt/wt}$, n = 4) and AlbCre^{+}STING KO (*AlbCre*$^{+/-}$*Tmem173*$^{-/-}$, n = 4) mice were i.v. infected with 5×10^{5} pfu WT MCMV. Blood was analyzed for IFN-β levels at **a** 4 hpi and **b** 36 hpi by an ELISA method. Data were retrieved in one experiment. Dashed line (DL= Detection Limit). Error bars indicate mean ± s.e.m. (*$p \leq 0.0286$; *ns* = not statistically significant; a two-tailed Mann-Whitney test was used to calculate p-values). Source Data are provided as a Source Data file.

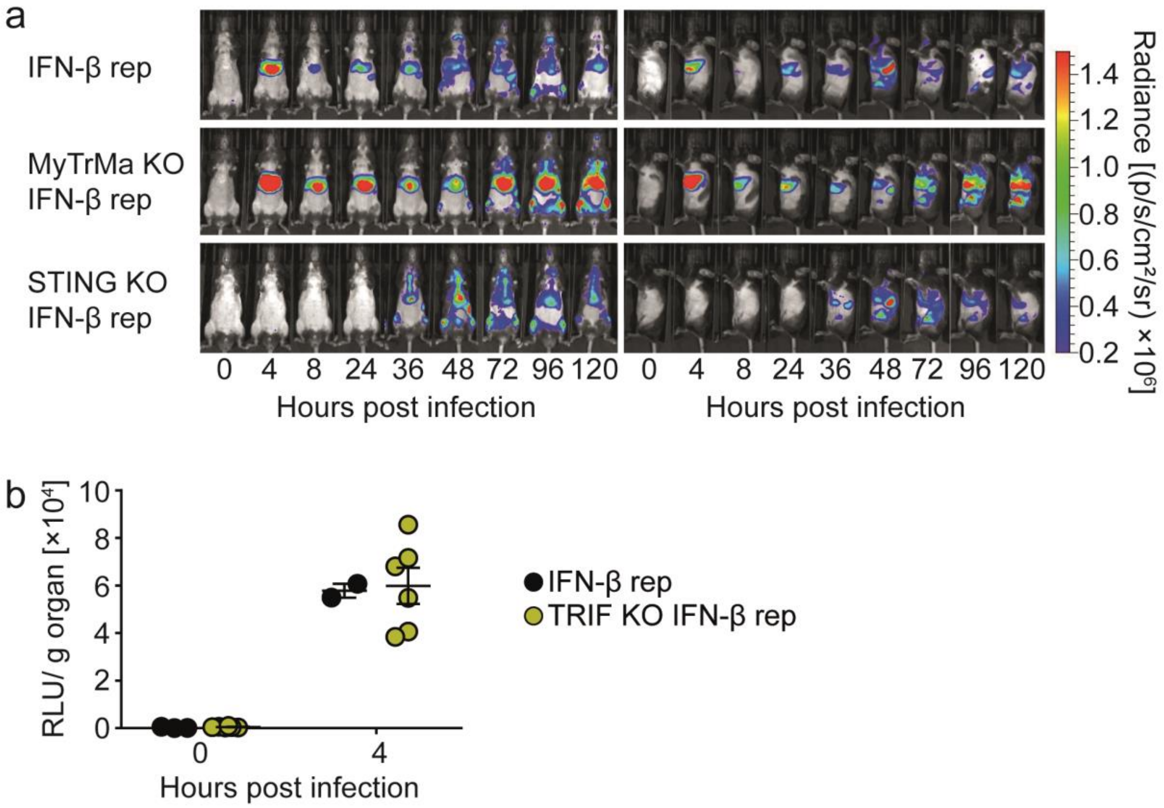

Supplementary Figure 3: STING signaling controls early hepatic IFN-β induction independent of TRIF signaling.

a IFN-β reporter (rep) (*Ifnb*$^{wt/\Delta\beta\text{-}luc}$, *n* ≥ 8), MyTrMa KO IFN-β reporter (*Myd88*$^{-/-}$*Trif*$^{-/-}$*Mavs*$^{-/-}$*Ifnb*$^{wt/\Delta\beta\text{-}luc}$, *n* ≥ 7), and STING KO IFN-β reporter (*Tmem173*$^{-/-}$*Ifnb*$^{wt/\Delta\beta\text{-}luc}$, *n* ≥ 8) mice were i.v. infected with 5 × 10^5 pfu MCMV Δ*m157*. At the indicated time points luciferin was i.v. injected and luciferase activity was monitored by *in vivo* imaging. One representative mouse out of at least seven similar ones is shown. **b** IFN-β reporter (*n* ≥ 2) and TRIF KO IFN-β reporter (*Trif*$^{-/-}$*Ifnb*$^{wt/\Delta\beta\text{-}luc}$, *n* = 6) mice were i.v. infected with 5 × 10^5 pfu MCMV Δ*m157*. The liver was removed, lysed and the bioluminescence, measured in relative light units (RLU), was quantified *in vitro*. Data are from at least two independently performed experiments. Error bars indicate mean ± s.e.m. Source Data are provided as a Source Data file.

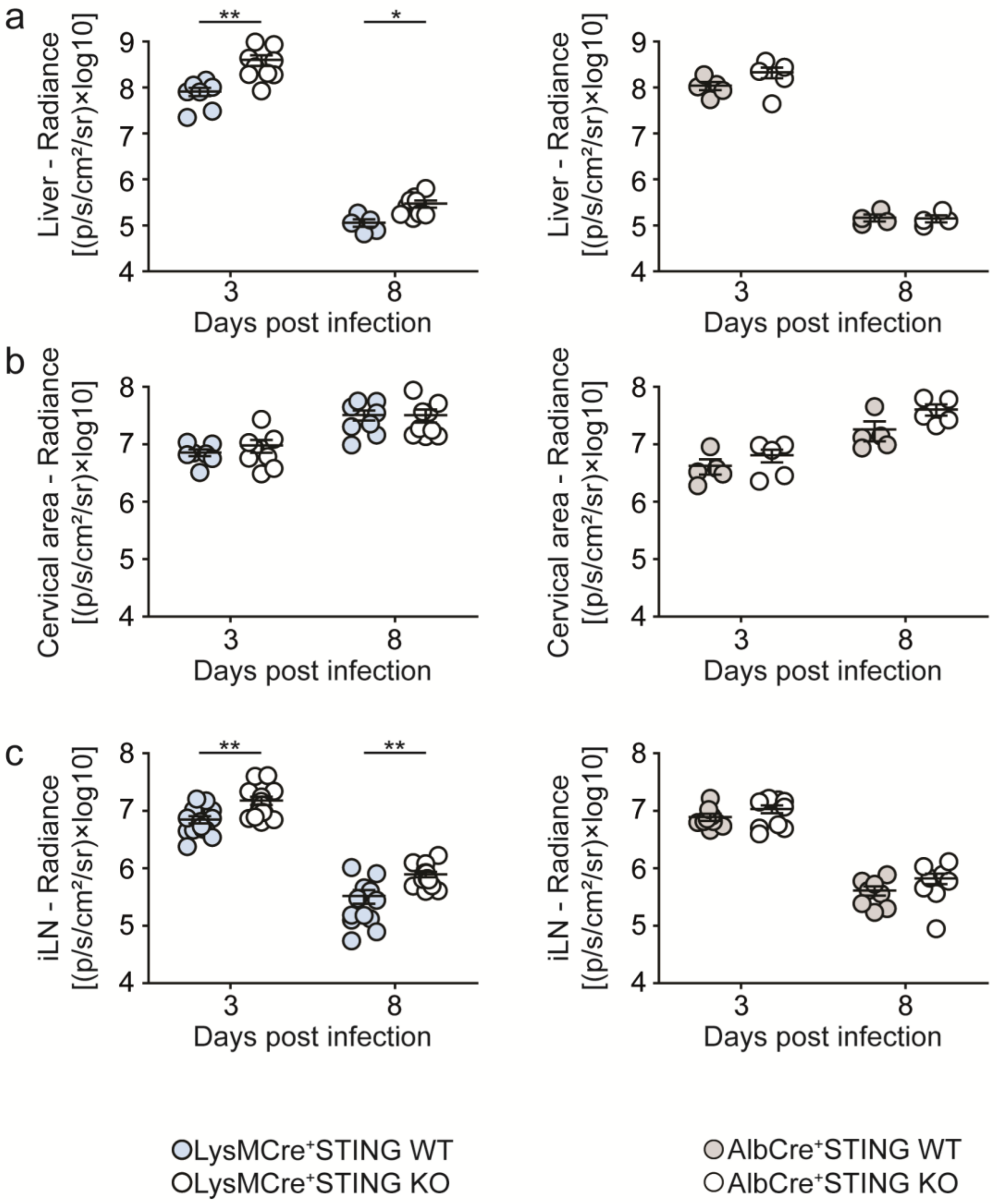

Supplementary Figure 4: STING signaling is important to control MCMV Δ*m157* propagation in the liver and in lymph nodes.

LysMCre$^+$STING WT (*Lyz2Cre*$^{+/-}$*Tmem173*$^{wt/wt}$, $n \geq 5$), LysMCre$^+$STING KO (*Lyz2Cre*$^{+/-}$*Tmem173*$^{-/-}$, $n = 9$), AlbCre$^+$STING WT (*AlbCre*$^{+/-}$*Tmem173*$^{wt/wt}$, $n \geq 4$), and AlbCre$^+$STING KO (*AlbCre*$^{+/-}$*Tmem173*$^{-/-}$, $n \geq 4$) mice were i.v. infected with 5×10^5 pfu MCMV Δ*m157luc*. At the indicated time points luciferin was i.v. injected and luciferase activity was monitored by *in vivo* imaging. **a** Liver, **b** the cervical area, and **c** inguinal lymph nodes (iLN) were marked as region of interest and bioluminescence signals were quantified. Data are from one or more experiments. Error bars indicate mean ± s.e.m. (*$p \leq 0.012$, **$p \leq 0.0013$; a two-tailed Mann-Whitney test was used to calculate *p*-values). Source Data are provided as a Source Data file.
